# Editorial: Application of Omics Approaches to the Diagnosis of Genetic Neurological Disorders

**DOI:** 10.3389/fneur.2021.712010

**Published:** 2021-07-07

**Authors:** Andrea Legati, Edoardo Giacopuzzi, Marco Spinazzi, Monkol Lek

**Affiliations:** ^1^Unit of Medical Genetics and Neurogenetics, Fondazione Istituto di Ricovero e Cura a Carattere Scientifico (IRCCS) Istituto Neurologico Carlo Besta, Milan, Italy; ^2^Wellcome Centre for Human Genetics, University of Oxford, Oxford, United Kingdom; ^3^National Institute for Health Research Oxford Biomedical Research Centre, Oxford, United Kingdom; ^4^Centre de Référence des Maladies Neuromusculaires, Centre Hospitalier Universitaire d'Angers, Angers, France; ^5^Department of Genetics, Yale School of Medicine, New Haven, CT, United States

**Keywords:** omics, neurological disorder, genetics, next generation sequencing, metabolomics (omics), transcriptomics, genomics, diagnosis

Neurological disorders comprise a huge variety of complex diseases, affecting the nervous systems and the skeletal muscle, which represent major causes of death and disability worldwide. Variants in a rapidly expanding number of genes involved in a broad variety of cellular functions are causally linked to a significant proportion of neurological disorders, acting alone or in combination with environmental factors. Over the past few years, the application of the different high-throughput approaches, also known as omics approaches (e.g., genomics, transcriptomics, proteomics, and metabolomics), has developed rapidly and revolutionized both the diagnosis and the understanding of the pathophysiology of neurological disorders, by providing un unbiased identification of disease-causing gene variants, biomarkers, and targets for potential therapeutic strategies. Omics techniques have been widely used in the past to research the genetic and molecular basis of neurological disorders. Today, given their rapidly decreasing costs, the increasing standardization of protocols, and the rapid turn-around time, these technologies are emerging also as a powerful addition to standard diagnostic procedures.

In this Research Topic four original papers highlight the potentialities of omics approaches in the diagnosis of genetic neurological disorders.

One of the most powerful diagnostic tool based on Next Generation Sequencing (NGS) is represented by targeted gene panels. Targeted re-sequencing using custom-made panels is highly efficient for the screening of up to hundreds potentially clinically relevant genes. This approach allows to perform genetic analysis in large cohorts of patients with affordable costs within a short timeframe, with the advantage of generating relatively low amount of data and easy to interpret results. In this Research Topic, to highlight the importance of targeted gene panels in the genetic diagnosis of neurological disorders, a study based on the genetic screening of more than 1,000 patients affected by autosomal optic atrophy have been included (Charif et al.).

Going beyond gene panels, a Whole Exome Sequencing (WES) approach allows the sequencing of all exons and exon-intron boundaries for all protein-coding genes in the genome. Its massive application in the last decade has dramatically increased the pace of disease gene discovery. The extensive, unbiased approach of WES usually lead to identification of around 20,000 coding variants per individuals, thus the analysis of results requires more advanced and complex strategies compared to targeted panel NGS. Often the interpretation of WES results needs to be integrated with additional available information such as clinical, metabolic, neuroimaging and biochemical data. However, WES approach can represent a powerful diagnostic tool in familiar cases, especially when a detailed clinical description and DNA samples from multiple family members are available. This is demonstrated by the case report added in our Research Topic, in which WES successfully identified a disease causing variant in a consanguineous family affected by spastic quadriplegia (Elsayed et al.).

In order to better characterize hidden genotype-phenotype correlations, it is crucial to develop integrative approaches that combine biological information coming from different sources to highlight the interrelationships of the involved biomolecules and their functions. This strategy can provide insights in the understanding, the diagnosis, the treatment of neurological diseases, and explore the boundaries of personalized medicine. For example, extracting and integrating heterogeneous information from neuroimaging and genomics can contribute to better define the correlation between neurological presentations and genetic alterations, improving the diagnostic process for several pathological conditions affecting the nervous system. This type of approach has been performed for the diagnosis of neuronal intranuclear inclusion in an original research article included in this Research Topic (Pang et al.).

In addition to genomics, also proteomics technology offers great promise in the understanding and treatment of the molecular basis of neurological diseases. Pathological conditions are the result of protein dysfunctions resulting from alterations of protein structure, abundance, activity, and/or interactions. The fast growing field of clinical proteomics aims to decipher protein changes associated to a specific disease, thereby providing ideal biomarkers for diagnosis, prognosis, and prediction of therapeutic response. Mainly based on its enormous hypothetical relevance, clinical proteomics is an ever-growing field. In the present Research Topic we included an original research article describing the use of proteomics for the identification of new potential biomarkers associated with autosomal recessive spastic ataxia of Charlevoix-Saguenay (ARSACS) (Morani et al.).

Pathogenic variants discovered in research cohorts of genetic diseases are ultra-rare and usually private to a family, highlighting the need to ensure that non-European populations are included in gene discovery efforts. Furthermore, it also emphasizes the need to use population specific allele frequencies to ensure variants are interpreted in the correct context. This Research Topic was diverse not only in genomic technologies outlined above but also included studies from Chinese (Pang et al.) and Sudanese (Elsayed et al.) populations.

This Research Topic would not have been possible without the contributions of several people. We would like to thank the authors of the submitted manuscripts for their precious studies and valuable data. We also thank the staff of Frontiers in Neurology for their dedicated assistance and support.

## Author Contributions

AL, EG, MS, and ML had the role of guest associate editors for the Research Topic entitled Application of Omics Approaches to the Diagnosis of Genetic Neurological Disorders. All authors contributed to the article and approved the submitted version.

## Conflict of Interest

The authors declare that the research was conducted in the absence of any commercial or financial relationships that could be construed as a potential conflict of interest.

